# Neurological Symptoms in Palliative Care Patients

**DOI:** 10.3389/fneur.2018.00275

**Published:** 2018-04-25

**Authors:** Johanna Anneser, Victoria Arenz, Gian Domenico Borasio

**Affiliations:** ^1^Palliative Care Team, Department of Psychosomatic Medicine and Psychotherapy, Klinikum rechts der Isar, Technical University Munich, Munich, Germany; ^2^Service de Soins palliatifs et de support, Centre Hospitalier Universitaire Vaudois (CHUV), University of Lausanne, Lausanne, Switzerland

**Keywords:** palliative care, neurological symptoms, prevalence, symptom burden, quality of life

## Abstract

**Background:**

Neurological expertise in palliative care may be required not only for patients with primary neurological disorders but also for patients with non-neurological diseases suffering from burdensome neurological symptoms. The aim of this study was to determine the prevalence of neurological diagnoses and symptoms in palliative care patients, as well as the related burden and impact on everyday life.

**Methods:**

We analyzed retrospectively the medical records of 255 consecutive patients from a tertiary medical center, at the time point of referral to an inpatient palliative care consultation service. In addition, 100 patients prospectively answered a questionnaire which included the assessment of neurological symptoms, as well as numeric rating scales for quality of life, symptom-specific burden, and restrictions in everyday life.

**Results:**

Forty-one patients (16%) suffered from a primary neurological disease. Most decisions regarding the termination of life-sustaining measures concerned this group (20/22, 91%). Neurological symptoms (excluding pain) were documented in 122 patients (48%) with an underlying non-neurological disease. In the questionnaire study, 98/100 patients reported at least one neurological or neuropsychiatric symptom, most frequently sleeping problems (*N* = 63), difficulty concentrating (*N* = 55), and sensory symptoms (*N* = 50). Vertigo/dizziness (*N* = 19) had the greatest impact on everyday life (7.57/10 ± 2.17) and the highest symptom-specific burden (7.14 ± 2.51). Difficulty concentrating (restrictions in everyday life/burden) and pain intensity were the only symptoms significantly correlated with quality of life (*r* = −0.36, *p* = 0.009/*r* = −0.32; *p* = 0.04; *r* = −0.327, *p* = 0.003).

**Conclusion:**

Neurological diseases and symptoms are frequent among palliative care patients and are often associated with a high symptom burden, which may severely affect the patients’ lives. It is thus of paramount importance to implement neurological expertise in palliative care.

## Introduction

Palliative care aims to provide physical, psychosocial, and spiritual care for terminally ill patients and their families. Although differences between countries exist, palliative care has been linked traditionally to oncological diseases and internal medicine/oncology specialists still constitute the largest part of the palliative care workforce in many countries (e.g., Germany, USA, Japan, and Canada) (1–4), while neurologists constitute less than 3% of physicians that are certified in palliative care (1, 2, 4). On the other hand, neurological and neurosurgical diseases have been found to be the second most common conditions in patients seen by a palliative care inpatient consultation service (5) and the most common diagnostic group in patients with non-malignant diseases (6).

Several reviews advocate a better integration of palliative care in the overall care of patients with neurological diseases (7–9). Both neurological and palliative care expertise are required, e.g., for patients with intracranial processes in order to assess prognosis, to substantiate decisions on the withholding or withdrawal of life-sustaining measures and to help the family understand the nature of an imminent persistent vegetative state or brain death. Although care concepts for people with motor neuron diseases may be an encouraging example of the successful integration of palliative care and neurology, many patients with neurodegenerative, neuro-oncological as well as neurovascular diseases still have a wide range of unmet palliative care needs (9, 10).

In addition, neurological expertise is required for non-neurological palliative care patients suffering from neurological symptoms. Scarce data are available on the prevalence of neurological symptoms in a general palliative care population and most of the data have been assessed retrospectively. In a meta-analysis looking at symptom prevalence in a total of 25,000 patients with incurable cancer (11), “neurological symptoms” (without further specification) were documented in 15% of all patients and in 34% within their last 2 weeks of life. Even less is known about the relevance of neurological symptoms for the patients’ lives (e.g., their subjective burden, the impact on patients’ quality of life (QoL), or possible restrictions in the patients’ everyday lives). We therefore combined a review of patient charts with a prospective questionnaire study in order to gain an overview of the frequency of neurological symptoms as well as their clinical relevance in a general palliative care population.

## Materials and Methods

### Retrospective Chart Analysis

The charts of 255 consecutive patients who had been referred to the palliative care inpatient consultation service, were analyzed. Data were collected over a period of 9 months. Demographic data, diagnosis, reasons for current admission to the hospital, previous treatments as well as neurological and neuropsychiatric symptoms and preconditions—as listed in the patient charts prior to the referral to the palliative care team—were assessed.

### Prospective Questionnaire Study

A total of 100 consecutive inpatients, referred to the palliative care inpatient consultation service, who were willing to participate and able to give their written informed consent were included in the questionnaire study. The questionnaire was administered in the form of a structured interview. The specific symptom burden is the degree to which the patients’ everyday lives were compromised by a symptom and the patients’ overall QoL were assessed using 0–10 point numeric rating scales (NRSs). NRS is a validated measure for QoL and is considered to assess the patient’s general QoL rather than only its health-related aspects (12).

In order to differentiate lightheadedness or pre-syncopal syndromes from vertigo/dizziness, we asked the patients for the sensation of spinning/swaying and/or unsteady gait.

Actual pain intensity was assessed using a visual analog scale (VAS). To be able to compare the symptom “pain” with other neurological/neuropsychiatric symptoms, we assessed also the symptom-specific burden and restrictions in everyday life due to pain. Neuropathic pain was diagnosed by clinical assessment of the pain characteristics and the association with typical symptoms in the same area (e.g., tingling, numbness).

The “Confusion Assessment Method” (CAM) questionnaire was used when delirium was suspected. The CAM is a validated instrument for the diagnosis of delirium in a variety of medical settings (13, 14).

The Patient Health Questionnaire (PHQ-4) was used to screen patients for anxiety and depression.

In addition, patients were asked to name the symptoms, which were currently most distressing to them (max. 5). All patients who agreed received a structured neurological examination. Diagnostic workup of burdensome neurological symptoms was offered if indicated (e.g., neuro-otologic tests in patients with vertigo/dizziness). In addition, all patients were offered psychological support if desired. Descriptive statistics was performed using SPSS 24 (IBM, Chicago, IL, USA). Correlation analysis (Spearman’s rho) was performed. The study was carried out in accordance with the recommendations of the Ethics Committee of the Technical University Munich. The protocol was approved by the Ethics Committee of the Technical University Munich (No: 5682/13). All subjects gave written informed consent in accordance with the Declaration of Helsinki.

## Results

### Analysis of Patient Charts

#### Reasons for Admission to the Hospital as Documented in the Patient Chart

Patients from nine different departments had been referred to the palliative care inpatient consultation service. For patient characteristics, see Table 1. In 84 out of 255 patients (33%), the main reasons or one of the main reasons for admission to the hospital were neurological symptoms (other than pain). 28 patients (11%) had been admitted directly to the departments of neurology or neurosurgery. The presence of neurological symptoms and preconditions—as documented in the admission examination or discovered in other examinations during the hospital stay—was assessed. Patients were assigned to one of four groups. (10 patients remained unclassified due to incomplete data or unclear assignment):

(1) A neurological condition is the primary underlying disease (e.g., stroke, neurodegenerative disorder, primary brain tumor): 41 patients (16%)
(2) Neurological symptoms (excluding pain) present and presumably caused by the (non-neurological) underlying disease and/or due to treatment of that disease: 99 patients (39%)
(3) Neurological symptoms (excluding pain) present and presumably independent from the basic disease: 23 patients (9%)
(4) No neurological preconditions or symptoms documented: 82 patients (32%)

**Table 1:** Analysis of patients’ medical records: patients’ characteristics and neurological symptoms.

Patient characteristics	255 patients	
Gender	118 (45%) female	
Mean age	67 ± 13 years	
Mean duration of disease	35 ± 54 months	
Cancer diagnosis	217 (85%)	
Primary brain tumor or cerebral metastases	49 (19%)	
Previous surgery	138 (55%)	
Previous chemotherapy	157 (63%)	
Previous radiotherapy	100 (40%)	

**Neurological symptoms as documented in the patient chart**		

Central paresis	41 (16%)	Group 1: 25/41 (61%) Group 2: 12/99 (12%) Group 3: 4/23 (17%)

Seizures	27 (11%)	Group 1: 22/41 (54%) Group 2: 14/99 (14%) Group 3: 1/23 (4%)

Dementia	14 (6%)	Group 1: 10/41 (24%) Group 2: 0/99 (0%) Group 3: 4/23 (17%)

Confusion	47 (19%)	Group 1: 15/41 (37%) Group 2: 27/99 (27%) Group 3: 4/23 (17%)

Other CNS symptoms	66 (26%)	Group 1: 33/41 (80%) Group 2: 27/99 (27%) Group 3: 6/23 (26%)

Peripheral paresis	27 (11%)	Group 1: 3/41 (7%) Group 2: 20/99 (20%) Group 3: 4/23 (17%)

Sensory symptoms	23 (9%)	Group 1: 0/41 (0%) Group 2: 20/99 (20%) Group 3: 3/23 (13%)

*Group 1: primary neurological condition, group 2: neurological symptoms presumably caused by a non-neurological underlying disease, and group 3: neurological symptoms presumably independent from the underlying disease*.

In most cases, no detailed neurological medical history had been assessed and no in-depth neurological examination had been performed before admission to the palliative care team. The most frequent neurological symptoms mentioned in the patient files are shown in Table 1. Neuropsychiatric symptoms such as sleeping problems, difficulty concentrating and impaired memory had not been assessed on a regular basis. “Taste abnormalities” were documented in none of the patient records.

#### Termination of Life-Sustaining Measures

After palliative care consultation, life-sustaining measures had been terminated in 22 patients, and 20 (91%) of them had been classified in group I (neurological condition as primary disease).

### Patient Questionnaire Study

#### Patient Characteristics

For the questionnaire study, 100 of 255 consecutive patients who were willing to participate and able to give their informed consent were included (50% female; 98 patients with cancer, 13 of them with brain metastases). 73 of 255 were not able to give their informed consent, 39 patients declined to participate, 32 patients could not be approached before the involvement of the palliative care consultation service, 11 lacked sufficient knowledge of the German language. None of the 41 neurologic patients could be recruited (20 were unconscious, 15 patients—mostly severe stroke or advanced glioblastoma—were conscious, but unable to consent, 5 refused to participate, and 1 lacked sufficient knowledge of the German language). 98 patients reported at least one neurological or neuropsychiatric symptom excluding pain, 38 patients stated that a neurologist or psychiatrist had treated them before admission to the hospital. Clinical neurological examination was performed after the patient interviews and objectified the symptoms reported (e.g., extent and degree of paresis or paresthesia). The values for “restrictions in everyday life” and “burden due to a specific symptom” were correlated, except for taste abnormalities.

#### Prevalence of Neurological Symptoms

The prevalence of neurological/neuropsychiatric symptoms, the symptom-specific burden, and restrictions in everyday life in patients participating in the questionnaire study is shown in Table 2. Sleeping problems (63/100), difficulties concentrating (55/100), and sensory symptoms (50/100) occurred most frequently. In addition, several neurological/neuropsychiatric symptoms were ranked among the most burdensome overall symptoms (Table 3).

**Table 2:** Prevalence of symptoms, restrictions in everyday life and burden due to specific symptoms (NRS 0–10).

Neurological symptoms	*n* = 100	Restrictions in everyday life Mean (±SD)	Burden due to a specific symptom Mean (±SD)
Pain	84	6.52 (±3.04)	6.48 (±3.08)
Sensory symptoms (numbness, tightness, tingling, burning)	50	4.67 (±3.27)	4.21 (±3.34)
Taste abnormalities	32	3.62 (±2.79)	5.62 (±2.83)
Hearing impairment	28	3.69 (±3.28)	3.85 (±3.16)
Muscular symptoms other than paresis (cramps, fasciculation)	28	NA	NA
Vertigo/dizziness	19	7.57 (±2.17)	7.14 (±2.51)
Paresis	16	6.08 (±3.36)	6.06 (±3.2)
Coordination difficulties	12	6.58 (±3.26)	5.75 (±3.08)
Double images	10	6.00 (±3.2)	5.90 (±3.38)
Seizures	6	4.75 (±4.43)	5.00 (±4.08)
Speech disorders	4	4.00 (±3.65)	6.50 (±3.11)

**Neuropsychiatric symptoms**			
Sleeping problems	63	5.70 (±2.93)	5.64 (±3.34)
Difficulty concentrating	55	3.85 (±3.18)	4.44 (±3.12)
Impaired memory	44	3.36 (±3.21)	4.29 (±3.11)

**Symptoms with possible neurological causes**			
Bladder or bowel disorder	30	2.56 (±3.00)	3.90 (±3.56)
Dysarthria and/or dysphagia	22	6.27 (±3.10)	6.45 (±2.96)

**Table 3:** Most distressing symptoms at the time of the interview named by 100 palliative care patients.

Frequency of mention	Symptoms
43×	General weakness, **pain**
20×	Dyspnea
13×	Nausea, fatigue, loss of appetite
9×	**Being worried/rumination**
5×	**Paresis**
4×	Loss of autonomy, social isolation, bleeding, **concentration problems, dysphagia**
3×	**Sensory symptoms, vertigo/dizziness**, diarrhea
2×	Problems with stoma care, **dysarthria, unsteady gait, anxiety, vision disturbances** fever
1×	Restlessness, cough, **sleep disturbances**, ascites, edema

*Neurological and neuropsychiatric symptoms as assessed in the questionnaire study are printed in bold*.

#### Neurological Symptoms and QoL

The mean QoL (±SD) (NRS 0–10) of all patients was 3.7 (±2.3). The only neurological non-pain symptom significantly correlated to QoL was difficulty concentrating (restrictions in everyday life: *r* = −0.36, *p* = 0.009; symptom-specific burden: *r* = −0.32; *p* = 0.04). In addition, the sum scores (restrictions in everyday life and symptom-specific burden) comprising the three most frequent neurological/neuropsychological symptoms (sleeping problems, difficulty concentrating and sensory symptoms) were significantly correlated with QoL (*r* = −0.348, *p* = 0.0004; *r* = −0.322, *p* = 0.001).

#### Pain

52 patients reported having only one pain localization, 27 patients reported having two, and 5 reported having three pain localizations. The most frequent type of pain was predominantly nociceptive (45 patients), followed by mixed pain (29 patients), and predominantly neuropathic pain (10 patients). Actual pain intensity—as measured by a VAS—correlated significantly with QoL (*r* = −0.327, *p* = 0.003). Restrictions in everyday life and subjective burden due to pain did not correlate significantly with patients’ QoL (*r* = −0.135, *p* = 0.21; *r* = −0.07, *p* = 0.517).

#### Depression and Anxiety (PHQ-4)

Normal scores (0–2) were found in 28 patients, mild distress (3–5) in 28 patients, moderate (6–8) in 23, and severe distress (9–12) in 21 patients. PHQ-4 showed a moderate correlation with QoL (*r* = 0.271, *p* = 0.007). The PHQ-4 depression subscore was significantly correlated with the neuropsychological symptom “difficulty concentrating” (*r* = 0.361 *p* = 0.003) and moderately, but significantly correlated with QoL (*r* = –0.271, *p* = 0.007).

#### Delirium

“Confusion” was documented in 47 patient charts (18.4%), 12 were able to give their informed consent to study enrollment and underwent a screening test for delirium (CAM). The test-specific criteria for the diagnosis of “delirium” were fulfilled in 11 patients. No significant correlation with regard to age or gender was found in our study group.

## Discussion

Several studies investigated the presence of burdensome symptoms in patients with advanced cancer and palliative care patients [for a review, see Ref. (11, 15)]. Isolated neurological symptoms such as agitation/delirium or vertigo/dizziness have been assessed in this context. However, a systematic and detailed analysis of neurological symptoms in palliative care patients has not been carried out before. We found that almost half of the palliative care patients without a primary neurological disease had neurological symptoms documented in their charts, and 98% of patients in the prospective study reported at least one burdensome neurological/neuropsychological symptom. This is considerably higher than previously described (11). These findings have an immediate clinical relevance in that significant suffering arises from symptoms, which are underdiagnosed and therefore insufficiently treated. Our data suggest that this may be the case for a variety of neurological symptoms, which come with a high subjective burden and/or restrictions in everyday life, e.g., vertigo/dizziness, coordination difficulties, or double vision (Table 2).

### Sensory Symptoms

The prevalence of sensory symptoms (numbness, tightness, tingling, and burning) reported in patients with advanced cancer and in palliative care patients varies widely from 6 to 36% (16–19). These variations may be caused by the different characteristics of the populations investigated, but also by the differing assessment methods used. Not surprisingly, it has been found that the prevalence of many symptoms is considerably lower when assessed using medical records as compared with studies using questionnaires or structured interviews (11). Correspondingly, the prevalence of sensory symptoms in our questionnaire study (50%) was markedly higher than the results from the chart reviews (9%).

### Dysgeusia

Similar results were obtained for the prevalence of “taste abnormalities” (dysgeusia) in palliative care patients: in our study, the occurrence of dysgeusia in the patients’ charts was 0%, while 32% of patients in the questionnaire study reported taste abnormalities. Similarly, a previous interview study found that 86% of palliative care patients had taste abnormalities (20), while dysgeusia was documented in only 1–2% of patients’ charts (17, 18).

### Dizziness/Vertigo

Interestingly, our questionnaire study revealed “dizziness/vertigo” as the most burdensome symptom with the greatest impact on everyday life. “Vertigo”—defined as an erroneous sense of motion and unsteadiness—is a relatively common condition, which also occurs in the general population: a survey in Germany reported a 12-month prevalence of 22.9% (21), while the point prevalence in palliative care patients with cancer was 10% (18). “Dizziness” is a term mostly used in a wider sense, which includes symptoms that range from a vague feeling of unsteadiness to severe vertigo. Pooled prevalence of dizziness in patients with incurable cancer was 17% in a previous study (11) and is similar to the occurrence found in our study (19%). Our questionnaire allowed only an initial screening for dizziness/vertigo without providing an accurate diagnosis. A detailed analysis of dizziness/vertigo in palliative care patients is urgently warranted, since effective treatment is available for many forms of this symptom (22).

### Pain

Pain had a prevalence of 84% in our palliative care population with a high symptom-specific burden and relevance for everyday life. Actual pain intensity correlated significantly with QoL, while symptom-related burden and restrictions in everyday life due to pain did not. An association between pain and QoL has been described previously, using the “brief pain inventory” (23, 24), but also an NRS (25). In contrast to the VAS assessing actual pain intensity, our items asking for a “symptom-specific burden” or “restrictions in everyday life” implicate significant elements of personal judgment and/or coping and may therefore provide different results when correlated with QoL. 39% of our—predominantly oncologic—patients suffered from neuropathic pain. This matches the prevalence of neuropathic pain in cancer patients assessed in palliative care (43%) and hospice settings (35%) (26). A recent review revealed that neuropathic pain in cancer patients is often insufficiently treated because of “incorrect use of co-analgesics” (27).

### Delirium

In 47 (19%) patient charts from our cohort, “confusion” was documented. This percentage matches previous observations in a palliative care population (28). Since patients had to give informed consent to participate in the prospective study, only 12 patients underwent CAM testing in the context of our study, which was positive in 11 patients. Prevalence, diagnosis, treatment, and prognosis of confusion/delirium in palliative care patients have been widely studied (29). Recently, a randomized controlled trial showed that palliative care patients treated with standard neuroleptic medication had more delirium-specific symptoms, more side effects and a shorter survival rate than placebo-treated patients (30). This underscores the necessity of a careful diagnostic evaluation and individualized management of delirium in palliative care. Expertise in neuropharmacology and in the early detection of extra-pyramidal side effects of neuroleptic medication may be helpful to tailor treatment for these patients.

### Anxiety and Depression

Anxiety and depression are common symptoms in palliative care patients (31) with an estimated prevalence ranging between 7 and 49% (32). Correspondingly, 21% of our patients scored 9–12/12 in the PHQ, indicating severe psychological distress.

### Sleeping Problems

Similar to previous studies (33–35), sleeping problems were a frequent complaint among palliative care patients with a relatively high symptom-specific burden and impact on everyday activities. Sleep problems are multifactorial in many patients, although the moderate correlation with subjective burden due to pain suggests that inappropriate pain therapy may contribute to sleep disturbances in our patient population.

### Cognitive Symptoms

A frequent occurrence of mild-to-moderate self-reported cognitive symptoms, such as difficulty concentrating or memory disturbances, has been described previously in palliative care and cancer patients (16, 17). In addition, in a previous study, cancer outpatients named “difficulty concentrating” as one of the 13 top-ranked symptoms (36). Correspondingly, difficulty concentrating was the only symptom (except pain) significantly correlated with QoL in our study. A previous report showed that complaints of difficulty concentrating did not correlate with objective measurements of cognitive function in palliative care patients (37). In our study group “difficulty concentrating” correlated significantly with the PHQ-4 depression subscore. It has been shown previously that the prevalence of depression in palliative care patients and patients with advanced cancer is greater than in the general population (38). Since difficulty concentrating is a complaint which is frequently associated with depression (39), this symptom may be part of a coexisting depressive syndrome. However, cognitive impairment and difficulty concentrating may also be accompanying symptoms of CNS processes or side effects of radiation therapy [for review, see Ref. (40)]. Expertise in organic forms of cognitive impairment may be helpful for the diagnostic classification of these symptoms in order to initiate appropriate treatment.

### Neurological/Neuropsychological Symptoms and QoL

The significant correlations between the sum scores of the three most frequently reported neurological/neuropsychiatric symptoms (sleeping problems, difficulty concentrating, and sensory symptoms) and QoL, as well as the frequency at which neurological problems are reported to be one of the most distressing overall symptoms (Table 3), underscore the extent to which these symptoms may compromise the patients’ well-being. Many neurological symptoms that turned out to be burdensome in the questionnaire study had not been assessed prior to the referral to the palliative care team. This underlines previous findings that physicians frequently tend to focus on their specialized scope of practice rather than giving sufficient attention to burdensome (e.g., neurological) symptoms in severely ill patients (41).

### Patients With Primary Neurological Diagnoses

The percentage of patients with a primary neurological diagnosis (16%) in our retrospective cohort is higher than the proportions found in previous studies (9.2 and 8.8%) (5, 6); one of which however excluded patients with primary brain tumors (5). In our study, the overwhelming majority (91%) of decisions regarding termination of life-sustaining measures (e.g., terminal extubation, termination of artificial hydration, and nutrition) were taken in patients with primary neurological diseases. This is in line with the findings of Liu et al. (6), who showed that “eliciting goals of care” was the most frequent reason for palliative care consultations among neurological patients. In-depth knowledge of the course and prognosis of neurological diseases is indispensable when discussing treatment options with the patients’ families. Generally, there is an increasing awareness that many patients suffering from neurological diseases have palliative care needs (7, 8) and efforts have been made to improve the education of neurologists in this context (42).

### Neurological Expertise in Palliative Care

Given the high prevalence and the considerable burden of neurological symptoms in palliative care patients, as well as the relatively high percentage of patients with primary neurological disorders, it becomes evident that neurological expertise is crucial in palliative care. It can be fostered by increased neurological training in postgraduate palliative care education, as well as by recruiting neurologists in specialized palliative care centers. However, relatively few neurologists choose to abandon their primary specialty to concentrate on palliative medicine full-time. Clinical rotations of neurologists in palliative care teams may not only help to integrate neurological knowledge into palliative care, but also open up career options for young neurologists (2).

### Limitations

The single center design may limit the generalizability of the study. However, referral of patients from nine different departments of our university hospital resulted in a highly heterogeneous study population with a wide range of diagnoses. Our study was conducted in a tertiary medical center that has departments of neurology and neurosurgery. Therefore, the number of patients with primary neurological disorders may be higher than in less specialized hospitals. In the prospective cohort, 98% of patients suffered from cancer. The significance of the results from the questionnaire study may therefore apply primarily to oncological palliative care patients. Unfortunately, this also reflects the disproportionate prevalence of tumor patients in most specialized palliative care centers worldwide.

Similar to other centers (43, 44) patients with non-malignant diseases including primary neurological disorders were referred to our palliative care consult service only at a very late stage. These patients were unable to consent and could not recruited for the prospective study. Similarly, patients of all disease stages and neurological symptoms that affected their ability to consent (e.g., delirium) were excluded from the study. Hence, the prevalence of neurologic symptoms in a general palliative care population may be even higher and the symptoms more burdensome than found in our prospective study group.

Finally, the NRSs for the assessment of symptom-specific burden and for restrictions in everyday life used in our study had not been validated previously. However, NRSs allow for a direct comparison between specific neurological symptoms and help reducing the study burden for the severely ill patients to a minimum.

## Conclusion

A majority of palliative care patients in our study suffered from neurological symptoms in varying degrees, frequently causing considerable symptom burden and restrictions in everyday life. Some of these symptoms are not well documented in patient charts and may remain undiagnosed and untreated. In addition, the question of withdrawal of life-sustaining treatment is most frequently posed in patients with primary neurological diseases. In consequence, palliative care teams are confronted on a daily basis with complex neurological questions and burdensome neurologic symptoms. This underscores the importance of the neurological expertise in palliative care teams.

## Author Contributions

JA was involved in the conception and design of the study, the statistical analysis and interpretation of data, and drafted the manuscript. VA was involved in the design of the study, in the acquisition of data, and approved the final manuscript. GB was involved in the conception of the study, and critically reviewed, revised, and approved the manuscript.

## Conflict of Interest Statement

The authors declare that the research was conducted in the absence of any commercial or financial relationships that could be construed as a potential conflict of interest.
